# Do plantar calcaneal spurs make the plantar aponeurosis enthesis stronger? A biomechanical analysis

**DOI:** 10.1111/joa.70090

**Published:** 2025-12-17

**Authors:** Joanna Tomlinson, Kai Chun Li, Johann Zwirner

**Affiliations:** ^1^ School of Anatomy University of Bristol Bristol UK; ^2^ Department of Anatomy University of Otago Dunedin New Zealand; ^3^ Department of Oral Rehabilitation University of Otago Dunedin New Zealand; ^4^ Institute of Legal Medicine, University Medical Center Hamburg‐Eppendorf Hamburg Germany; ^5^ Department of Oral Sciences University of Otago Dunedin New Zealand

**Keywords:** avulsion parameters, calcaneus, enthesis, plantar spurs, pull‐out force

## Abstract

Calcaneal spurs are shown to be increasingly prevalent in modern populations and often contribute to forming heel and foot pain. There are multiple hypotheses for their formation, including exercise, prolonged standing and obesity. The impact of these spurs on foot biomechanics remains unclear; it is suggested that their presence may contribute to enthesial avulsion forces. This study aimed to determine the avulsion properties of the plantar aponeurosis enthesis with and without spurs. Twenty‐four feet from 15 cadavers donated to the Department of Anatomy at the University of Otago were used for this study. Tissues were X‐rayed to determine the presence of spurs. The donor feet were dissected to isolate the calcanei. These were then mounted in a custom‐developed 3D‐printed clamping rig to perform tensile testing of the plantar calcaneal enthesis to determine pull‐out forces of the central band of the plantar fascia. Biomechanical testing showed no statistically significant differences in avulsion properties between the spur (*n* = 7) and non‐spur (*n* = 14) samples in any of the avulsion parameters investigated: *F*_max_ (1121 ± 358 N vs. 953 ± 283 N, mean ± SD, *p* = 0.302) and *εF*_max_ (53 ± 11% vs. 51 ± 13%, mean ± SD, *p* = 0.660). Despite this, the avulsion parameters were highly variable. The results of this study indicate that the pull‐out force of the central band of the plantar fascia is unrelated to the presence of spurs. Therefore, it is less likely that plantar spurs fulfill a biomechanical function within the plantar fascia complex.

## Graphical abstract

Novel testing set up for determining enthesial avulsion parameters. Donor calcanei were dissected to isolate the central band of the plantar fascia, and mounted in a custom 3D‐printed rig (Fig A, B, C) for uniaxial tensile testing of the plantar fascia enthesis (Fig D). This study demonstrated no significant differences in the avulsion parameters, Fmax and εFmax, between spur and non‐spur specimens, indicating that plantar calcaneal spurs are unlikely to influence the enthesis’ biomechanics.

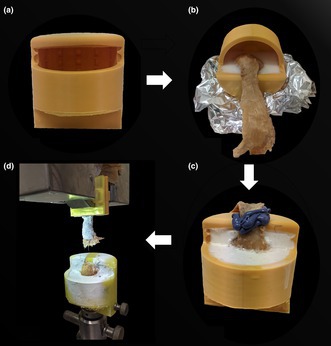

## 1 INTRODUCTION

Calcaneal foot spurs (calcaneal exostoses) are osseous outgrowths which are present either posteriorly in relation to the Achilles tendon (Rufai et al., 1995; Zwirner et al., 2021) or on the plantar aspect in relation to the plantar aponeurosis (also known as plantar fascia) (Plettner, 1900). Despite a large amount of research on the topic, it remains unclear to date whether calcaneal spurs present pathological findings or non‐pathological exostoses (Alatassi et al., 2018). Historic reports show that plantar calcaneal spurs are present in 11%–21% of individuals (Kirkpatrick et al., 2017), however, results from X‐rays of a modern population show that these are present in 32% of individuals (Beytemur & Oncu, 2018). The frequencies of these spurs appear to increase with age (Weiss, 2012). Dependent on location, these are thought to occur either due to exercise activities (posterior calcaneal) or be related to the modern phenomenon of standing for long periods of time and increasing rates of obesity (plantar calcaneal) (Weiss, 2012). In some cases, these are present along with co‐existence of plantar aponeurosis thickening; these lead to individuals exhibiting heel pain (Lee et al., 2023; Menz et al., 2019).

In line with Benjamin et al. (2000), it is hypothesized that soft tissue tension promotes the growth of enthesial fibrocartilage to form calcaneal spurs (Zwirner et al., 2021). This potentially leads to a stronger connection between the plantar fascia and Achilles tendon with the calcaneus, thus suggesting higher forces are required to avulse both their aponeuroses from the bone (Kumai & Benjamin, 2002; Li & Muehleman, 2007). In theory, the increase of the attachment area of the enthesis on the calcaneus through spur development could make the attachment stronger as a whole compared to a spur‐free, smaller attachment area. Alternatively, spurs are thought to form through repetitive loading from ground reaction forces following Wolff's law, causing micro‐trauma and alignment of the trabecular architecture (Kumai & Benjamin, 2002; Li & Muehleman, 2007), which continue to progress resulting in pain. It remains unclear how the presence of a plantar calcaneal foot spur impacts or contributes to the biomechanical functioning of the foot. Therefore, based on this information, the suitable mode of prevention, management or treatment of plantar calcaneal spurs remains unclear. This study therefore aimed to determine the avulsion properties (maximum tensile force, which refers to the maximum force before sample breakage (*F*_max_)) and strain at *F*_max_ (describing the strain of the sample at the point of the maximum tensile force) of the plantar aponeurosis enthesis at the calcaneus with and without plantar calcaneal heel spurs. It is hypothesized that the *F*_max_ of the plantar aponeurosis will be greater in specimens with plantar calcaneal heel spurs due to the potential larger surface area at the enthesis.

## 2 METHODS

### 2.1 Specimens

Twenty‐four feet from 15 Crosado‐embalmed donors were used for this study from the Department of Anatomy at the University of Otago (mean age ± SD: 82.3 ± 5.5 years; 1 female, 14 male donors, 10 right, 14 left feet) (Crosado et al., 2020). Ethical approval was granted by the Human Ethics Committee Board at the University of Otago, NZ (reference number: H20/071). Māori consultation was sought, and approval for this study was granted by the Ngāi Tahu Research Consultation Committee. Tissues that were visibly or potentially micro‐ or macroscopically altered by musculoskeletal degeneration or pathology, or the result of the embalming or dissection process, were excluded from this study.

### 2.2 Preparation of the experimental set‐up

#### 2.2.1 Preparation of testing clamps

**FIGURE 1:**
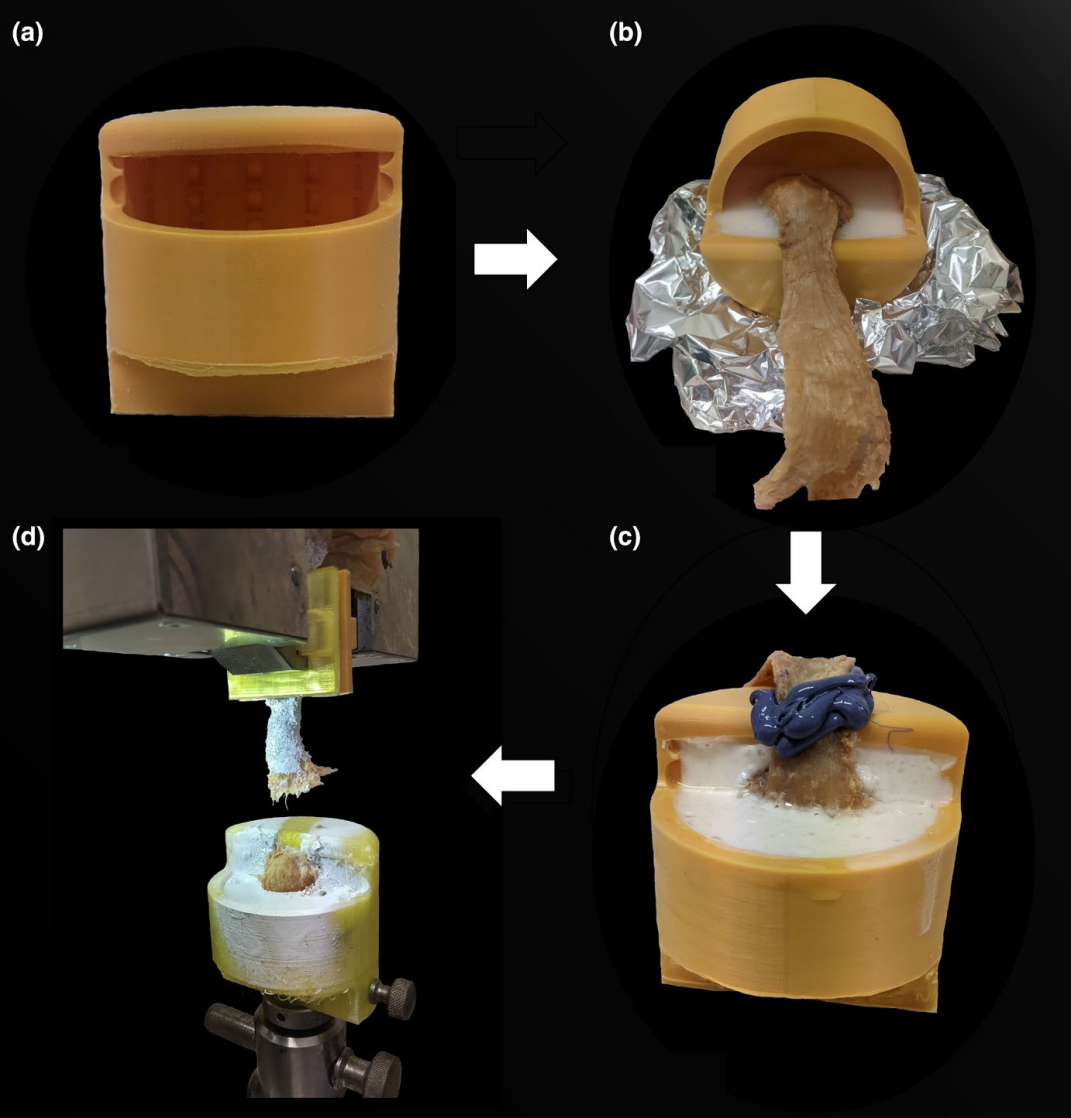
Methodology of tensile testing the avulsion forces of the plantar aponeurosis enthesis. (a) Printed inferior clamp for embedding the calcaneus. (b) Part one of the embedding process to secure the calcaneus in the clamp using resin (white). This was aligned centrally to fit against the clamping set up. (c) Part two of the embedding process whereby the resin was poured in a different orientation. Additionally, the cross‐sectional area was measured using (purple) moulding medium. (d) Plantar aponeurosis placed into clamping set‐up and stretched until failure.

Customized components to fix the central band of the plantar fascia to the upper jaw and the calcaneus to the lower jaw of the machine were designed using computer‐aided design software (Autodesk Inventor 2021, Autodesk, US) and manufactured with a commercial 3D printer (UltiMaker S3; UltiMaker B.V., Utrecht, The Netherlands) (Figure 1a). Two serrated polylactic acid clamps were printed, which connected to the upper jaw of the uniaxial universal testing machine (Model Z020, Allround Table Top with an Xforce P load cell of 2.5 kN; all Zwick‐Roell, Ulm, Germany). A 3D‐printed cup‐like shell‐testing clamp was printed in polylactic acid for embedding the calcaneus within and connecting to the lower jaw of the universal testing machine.

#### 2.2.2 Preparation of specimens

Prior to dissection, lateral X‐rays were obtained of the foot and ankle of each specimen using a Philips BuckyDiagnost Ceiling System (Philips, Amsterdam, The Netherlands) for assessment of calcaneal spurs. The tube voltage was 50 kV and the milliamperage was 4.00 mAs. The resulting exposure time was 15.1 ms. These X‐rays were then assessed by two investigators (JT and JZ) to determine the presence of calcaneal spurs, and their length was measured along the longest side that deviated from the calcaneus (from the border of the calcaneus to the tip of the spur) using Sectra Image Viewer (version 23.1.9.4561, SectraAB, Linköping, Sweden) and Microsoft PowerPoint (version 16.16.25, WA, USA). A calibration ball was used in images to standardize measurements and allow for calibration of measuring. Crosado embalmed (Crosado et al., 2020) donor feet were then dissected by an anatomist (JT) to remove the skin, subcutaneous fat, associated muscles and isolate the central band of the plantar aponeurosis and its attachment onto the calcaneus. The medial and lateral bands were removed, as the orientation of these fibres is different from that of the central band. Tissues were kept hydrated in an airtight bag with 0.9% saline solution. The anterior and superior parts of the calcaneus were removed with a rotating blade to allow it to fit within the testing clamp.

Specimens were then embedded within the testing clamp by pouring a solution of ceramic powder reinforced polyurethane resin in a two‐step process (Rencast FC52 Isocyanate/FC53 Polyol/Ceramic Powder; RenShape solutions, Huntsman International LLC, Salt Lake City, UT, USA) into the mould and placing the specimen in situ (Figure 1b,c). This was performed in a manner that spared the attachment region of the central band of the plantar fascia for the subsequent pull‐out experiments.

To obtain geometric measures of the tissues with the aim of understanding avulsion parameters, the cross‐sectional area (CSA) of the thinnest portion of the central band of the plantar aponeurosis was measured using a standard procedure described previously (Scholze et al., 2018) (Figure 1c). This process involves creating a mould around the thinnest portion of the plantar aponeurosis using polysiloxane impression material (medium‐bodied, Exahiflex; GC Corporation, Tokyo, Japan) (Figure 1c). As performed in previous research, the resulting mould was cut in half to allow the CSA to be measured based on an average of two individual area measurements, then being scanned on a commercial scanner (Perfection 7V750Pro; Seiko Epson Corporation, Suwa, Japan; 1200‐dpi resolution). A graphite powder stochastic speckle pattern was applied to the tissue for optical calibration and gathering strain data, which was shown previously not to influence the biomechanical properties of the human iliotibial tract, and therefore likely similar effects may occur with speckling plantar fascia tissue (Zwirner et al., 2020) (Figure 1d). This process allows for optical tracking of the surface deformation, with a camera (Q400, Limess, Krefeld, Germany; 2.8 megapixel resolution) to determine the maximum strain. The imaging set up was also used to obtain qualitative information relating to the failure site and errors with the testing set‐up.

### 2.3 Mechanical testing

Tissues were then clamped within the universal testing machine (Model Z020, Allround Table Top with an Xforce P load cell of 2.5 kN; all Zwick‐Roell, Ulm, Germany), then stretched until failure at a displacement rate of 20 mm min^−1^ and a sampling rate of 100 Hz (Figure 1d). This value falls outside the range of in vivo loading conditions of the plantar fascia during walking (Gefen, 2003). The testControl II (including real‐time compliance correction irrespective of test speed and duration and therefore accounted for in the data presented) was configured to tensile test the tissues and reported the maximum force (*F*_max_) and strain at maximum force (*εF*_max_) of the tissue at the enthesis. The specimens were visually inspected following tensile testing to determine the location of the rupture, which was either:

- Failure at the enthesis, where the enthesis is pulled off the bone and ruptures within the fibrocartilage–bone interface.
- Avulsion of the bone.
- Rupture within the fascia.

### 2.4 Statistics

The data were statistically analysed using Microsoft Excel (version 16.16.25, WA, USA) and SPSS (version 27.0, IL, USA). A Shapiro–Wilk test was used to test the Gaussian distribution of the data. *P* values of 0.05 or less were considered statistically significant. For unpaired parametric data, an independent sample *t*‐test was performed, and Pearson correlation was performed for determining the relationship between the spur length and avulsion parameters.

## 3 RESULTS

Of the available samples that underwent biomechanical analysis, the radiological analysis showed that nine of the specimens had plantar calcaneal spurs (mean ± SD; 82.9 ± 4.9 years; 2 female, 7 male feet; 4 right, 5 left feet) at the location of the plantar aponeurosis, 15 had no plantar calcaneal spurs (mean ± SD; 82.1 ± 6.1 years; 0 female, 15 male feet; 6 right, 9 left feet) (Table 1).

**TABLE 1:** Demographic and biomechanical data from cohort.

	Age	Specimen demographics			Biomechanical parameters	
		Sex	Side	Presence of spur?	*F*_max_	ε*F*_max_
1	84	M	L	No	Failed a clamp	Failed at clamp
2	82	M	L	No	540	46
3	82	M	R	No	1105	47
4	90	M	L	No	991	51
5	84	M	R	No	1167	55
6	76	M	R	No	1199	56
7	89	M	L	No	1085	85
8	89	M	R	No	1198	62
9	82	M	L	No	771	45
10	84	M	R	No	889	56
11	84	M	L	No	790	37
12	83	M	R	No	629	35
13	83	M	L	No	608	49
14	70	M	L	No	816	36
15	71	M	L	No	1548	53
16	78	M	L	Yes	Failed a clamp	Failed at clamp
17	78	M	R	Yes	Failed at clamp	Failed at clamp
18	84	F	L	Yes	771	49
19	84	F	R	Yes	1331	46
20	86	M	R	Yes	1403	60
21	76	M	L	Yes	1179	56
22	76	M	R	Yes	1170	63
23	88	M	L	Yes	505	35
24	86	M	L	Yes	1492	64

*Note*: Details are depicted.

Abbreviations: ε*F*_max_, strain at maximum force (%); F, female; *F*_max_, maximum force (N); L, left; M, male; R, right.

### Avulsion properties of the plantar calcaneal aponeurosis enthesis

Twenty‐four specimens were tested in this study; 21 of the specimens failed at the enthesis, resulting in avulsion of bone, three failed at the clamp (one without spurs and two with a plantar spur). As such, the avulsion parameters of the specimens were highly variable across the whole data set. The data of specimens which failed at the enthesis are presented in Table 2.

**TABLE 2:** The avulsion properties of the samples that avulsed from the bone are presented as mean ± standard deviation (median in parentheses) and *p*‐values of the independent samples *t*‐test statistical comparisons.

	Avulsion properties		Statistical comparison
	Plantar calcaneal spur (*n* = 7)	No spur (*n* = 14)	
*F*_max_	1121 ± 358 (1179)	953 ± 283 (940)	NS—0.302
*εF*_max_	53 ± 11 (56)	51 ± 13 (50)	NS—0.660

Abbreviations: *εF*_max_, strain at maximum force (%); *F*_max_, maximum force (N); NS, not significant (*p* > 0.050).

### 3.1 Morphological features and correlations to the measured biomechanical parameters

The CSA of the central band of the plantar fascia in the specimens was highly variable and the samples ranged from 45.65 to 106.38 mm^2^ for the whole data set, with a median of 65.53 mm^2^, mean of 66.66 mm^2^ standard deviation of 16.58 mm^2^. No significant difference was noted in the CSA of the central band of the plantar fascia between samples with and without spurs (74.70 ± 16.99 vs. 61.83 ± 14.83, *p* = 0.079; mean ± standard deviation).

The dimensions of the plantar calcaneal spurs as determined by X‐ray were varied in length (6.30–11 mm, 8.52 ± 1.74 mm; range, mean ± SD). They were consistently located at the anterior–inferior aspect of the calcaneal tuberosity. No significant correlation was noted between spur length and either *F*_max_ (*p* = 0.215, *r* = −0.492) or *εF*_max_ (*p* = 0.552, *r* = −0.249).

## 4 DISCUSSION

This study is the first to measure the enthesial force of the central band of the plantar aponeurosis, using a novel testing step up. This study presents that the avulsion forces of the central band of the plantar fascia from the calcanei in specimens with and without spurs were not different. It may be inferred that the enthesial strength in these specimens is likely similar. The plantar aponeurosis fibre orientation at the enthesis is in alignment with the trabecular architecture within the bone (Zwirner et al., 2021), which is shown to be connected to the subchondral bone via the enthesial fibrocartilage (Zwirner et al., 2021). This arrangement is in line with ligament and tendon enthesis (Benjamin et al., 2000), which act as an area of force transmission, stress concentration and anchorage (Benjamin et al., 2006). It has been suggested that spurs may function to adapt to stress, in a similar fashion to osteophyte formation (Benjamin et al., 2006). However, further work is required to determine the relationship with material properties, fibre orientation and loading conditions to ascertain the biomechanical significance of spurs.

### 4.1 Enthesial force is likely variable between individuals

The results provided herein show that the avulsion properties of the central band of the plantar aponeurosis calcaneal enthesis are highly variable. Although insignificant, there appears to be a moderate trend between *F*_max_ and spur length, suggestive that pull‐out forces of the plantar fascia are higher with shorter spurs. However, according to the results of this study, the presence of calcaneal spurs does not appear to influence the avulsion parameters of the enthesis, yet strain mapping techniques, such as digital image correlation (Luyckx et al., 2014), and a larger sample are required to confirm this. One interpretation of the *F*_max_ values being similar between entheses with and without spurs is that the spur development increases the pull‐out force of the plantar fascia compared to the spur‐free enthesis prior to the development of a spur through an increase in surface area. Thereby, the increased enthesial pull‐out force of the calcaneus derived from the spur may compensate for the pull‐out forces of the calcanei derived from deficits in the soft tissues prior to the development of spurs. Kumai and Benjamin (2002) propose that these deficits promote the development of spurs, thought to occur through endochondral ossification of fibrocartilage enthesis, and shown by Saito et al. (2022). These degenerative changes are induced by increased stress (Saito et al., 2022). This is also accompanied by fibrocartilage mineralization, collagen fibril fragmentation and upregulation of Col10, Runx2 and Mmp13 (Saito et al., 2022), which regulate chondrocyte proliferation and differentiation. Therefore, presumably, this compensation shows a well‐balanced ratio between the occurring forces on the enthesis and the forces the enthesis can withstand at maximum. Hence, after the spur development, the pull‐out forces between entheses with and without spurs are similar. However, if the aforementioned theory applies, the statistical similarity of the pull‐out forces would mask that this is an effect of an adaptation process. Moreover, the relationship between pull‐out force and attachment could be more complex. For example, it might be that some bony insertions are more efficient compared to others, meaning that the enthesis as a whole does not require a large attachment area. In other cases, the attachment might be weaker, which then could be balanced by increasing the attachment area and strengthening the insertion as a whole, and therefore, this phenomenon may not be observed at all entheses.

Further work is required to determine if other individual and local factors, such as age, weight, height and variations in micro‐ and macro‐scopic anatomy influence enthesial forces, spur formation and location of stress concentration. For example, previous research has suggested that fibre orientation is likely an important factor in mechanical properties (Golman et al., 2021). While research debates the role of muscles in defining enthesis structure in astronauts, it suggests skeletal muscle activation may contribute to enthesis remodelling and changes to compressive and tensile strength (Roffino et al., 2021). This hypothesis is supported by murine models of skeletal muscle repair (Wang et al., 2018), and evidence of adaption in response to muscle loading (Ganji et al., 2023), therefore, it may be postulated that human calcaneal shape, size and biomechanical properties may be adaptive. Plantar calcaneal spurs are also shown to arise at the insertion of the abductor digiti minimi and flexor digitorum brevis (Kirkpatrick et al., 2017), while atrophy of the abductor digiti minimi is also associated with heel pain (Chundru et al., 2008). This, therefore, highlights other morphological structures (abductor digiti minimi and flexor digitorum brevis anatomy) for future research focus to determine their relationship to presence, morphology and biomechanics of calcaneal spurs.

### 4.2 Morphology of enthesial spurs may contribute to biomechanical variance

Calcaneal spurs are known to vary in terms of the size (Cho et al., 2022; Okcu et al., 2023), slope (Sahin & Sabri Balik, 2023) and length (Sahin & Sabri Balik, 2023) of the spur, and these variables are related to pain. The results of this study show that the features of the calcaneal spurs were in line with previous literature (Kuyucu et al., 2015). However, data on donor medical history did not include detailed information on foot pain. Furthermore, other morphological parameters, such as the width, number of peaks, distribution of peaks, angulation of tapered processes and relative area of the spur, remain unknown, as is the specific attachment of the plantar aponeurosis, which may be inferior to the spur (Type A) or contained within the spur (Type B) (Velagala et al., 2022). It may be hypothesized that the surface area and concomitant biomechanical properties may vary between such cases. There was insufficient data available to determine if the avulsion properties altered according to spur length, surface area, slope, spur type or other factors, such as the concomitant presence of posterior and plantar spurs, which occurs in Achilles tendonitis (Zhu et al., 2020). Enthesial avulsion forces are likely part of a dynamic relationship with the spur characteristics, mediated by a multitude of factors, interlinked with the foot core complex, which is known to play a role in the stability and mobility of the foot. Future work should consider the type and morphometric properties of the plantar calcaneal spur and enthesis using three‐dimensional visualization to better understand its physiological role and better define the progressive classification of these structures. This information would be insightful to support investigations into the role of these parameters and the corresponding biomechanical properties to further elucidate the impact of calcaneal enthesis on the kinematics of the foot.

### 4.3 Limitations

The study is limited by numerous factors, firstly by the ex‐vivo conditions of methodology, which were required to ensure appropriate ethics and feasibility to assess the aims of this study. It cannot be excluded that the embalming fluid, dissection preparation and environmental conditions caused tissue damage, which might have lowered the reported pull‐out forces. However, recent research investigating the biomechanical properties of dura mater and iliotibial band found no change in *F*_max_ when comparing Crosado embalming to fresh samples (Tomlinson et al., 2025). Secondly, the study focused on the central band of the plantar fascia, as the main proportion of the plantar fascia contributing to the enthesis; different results may be produced if the fibres of the lateral or medial band were placed under testing conditions. Thirdly, the area of the enthesis was not measured in this study given that the enthesis was destroyed in testing, and it was unclear how the irregular shape of the spurs impacted the surface area of the enthesis. Future work should aim to address determining this across a cohort. However, it is important to note that CSA does not impact the calculation of *F*_max_. Fourthly, determining the location of spurs in the medio‐lateral plane of the calcaneal tuberosity could not be distinguished in the X‐ray images, and therefore, the footprint of the spur enthesis remains unclear. Fifthly, the study is limited by the sample size which was assessed; this was due to the number of cadaveric specimens that were available to be allocated to the given study. As a result, the results and subsequent conclusions of the work may not apply to individuals not included in the demographic representation of this study, that is, age or ethnicity. Moreover, the medical history of the donors was unknown. Equally, further individual nuances to avulsion parameters may be present within the data set, but are unable to be explored appropriately. Sixthly, given the small sample size, the strain rates applied were one uniform speed; however, it is acknowledged that tissues are strain rate dependent and will experience different strain rates in different scenarios, for example, running compared to walking. Also, the strain rate applied herein is not representative of those conditions (Gefen, 2003). Finally, quantitative data obtained from the image correlation set up were not used in this study, a parameter that may increase the accuracy of the findings, as it was deemed invalid because data from several experiments failed to be captured. This was due to the displacement of water during the experiment, which in turn displaced graphite powder speckling. The samples were considered appropriately hydrated as they were kept in plastic bags to retain moisture and also because the time taken from preparation to testing was <3 min. Therefore, water loss during preparation was deemed negligible. These limitations therefore restrict the clinical applicability of the results herein.

## 5 CONCLUSION

The presence of calcaneal spurs does not appear to alter the enthesial pull‐out force and maximum strain of the central band of the plantar fascia before pull‐out at its enthesis. Furthermore, the absence of such a relationship and the aforementioned study limitations suggest that the biomechanical and morphological characteristics of the central band of the plantar fascia and other associated tissues should be explored as they may mediate the presence of plantar spurs. Also, the absence of potential confounding variables in the cadaveric setup highlights that co‐contraction, weight distribution, and in vivo muscular influence may impact the biomechanics of the enthesis.

## AUTHOR CONTRIBUTIONS

This project was conceptualized and designed by Dr Johann Zwirner, Dr KC Li and Dr Joanna Tomlinson. Dr Joanna Tomlinson performed the acquisition of data, data analysis/interpretation and initial drafting of the manuscript. All authors contributed to the critical review of the manuscript and approval of the article.

## Data Availability

The data that support the findings of this study are available from the corresponding author upon reasonable request.
